# A Novel Relative Position Estimation Method for Capsule Robot Moving in Gastrointestinal Tract

**DOI:** 10.3390/s19122746

**Published:** 2019-06-19

**Authors:** Min Wang, Qinyuan Shi, Shuang Song, Chao Hu, Max Q.-H. Meng

**Affiliations:** 1 Shenzhen Engineering Lab for Medical Intelligent Wireless Ultrasonic Imaging Technology, Harbin Institute of Technolgoy, Shenzhen 518055, China; wangmin_hit@outlook.com (M.W.); 15562105085@163.com (Q.S.); 2 Ningbo Institute of Technology, Zhejiang University, Ningbo 315000, China; 3 Robotics, Perception and Artificial Intelligence Lab, The Chinese University of Hong Kong, N.T., Hong Kong 999077, China; qhmeng@ee.cuhk.edu.hk

**Keywords:** capsule robot, magnetic sensor array, relative distance, trajectory fitting, magnetic localization

## Abstract

Recently, a variety of positioning and tracking methods have been proposed for capsule robots moving in the gastrointestinal (GI) tract to provide real-time unobstructed spatial pose results. However, the current absolute position-based result cannot match the GI structure due to its unstructured environment. To overcome this disadvantage and provide a proper position description method to match the GI tract, we here present a relative position estimation method for tracking the capsule robot, which uses the moving distance of the robot along the GI tract to indicate the position result. The procedure of the proposed method is as follows: firstly, the absolute position results of the capsule robot are obtained with the magnetic tracking method; then, the moving status of the robot along the GI tract is determined according to the moving direction; and finally, the movement trajectory of the capsule robot is fitted with the Bézier curve, where the moving distance can then be evaluated using the integral method. Compared to state-of-the-art capsule tracking methods, the proposed method can directly help to guide medical instruments by providing physicians the insertion distance in patients’ bodies, which cannot be done based on absolute position results. Moreover, as relative distance information was used, no reference tracking objects needed to be mounted onto the human body. The experimental results prove that the proposed method achieves a good distance estimation of the capsule robot moving in the simulation platform.

## 1. Introduction

According to a report of the World Health Organization (WHO), cancer is considered as the leading cause of death worldwide [1], accounting for the deaths of nearly 10 million people every year. In terms of mortality, colorectal cancer (CRC) is the third highest among all cancers, and the number of deaths were about 774,000 in 2015. Gastrointestinal (GI) diseases, especially in the small intestine and colon, are extremely harmful to the public’s health. However, if it can be detected in its early stages and treated promptly, the survival rate could increase up to 94% [2].

Conventional endoscopes with cables is the most common tool used in examinations, but often bring pain to the patient during the examination. Moreover, this process requires narcotic drugs, which may be risky to the patient. In recent years, robot-assisted colonoscopy and the use of micro-robots in the GI tract have made significant progress [3,4,5]. Capsule robots, as a smart micro-tool, can enter the human GI tract for use in medical exploration and treatment. It causes minimal stimulation and damage to the human body, and is a new breakthrough in therapeutic medical technology and interventional therapy. After it is swallowed and enters into the GI track, it can photograph organs such as the oesophagus, stomach, small intestine, and large intestine, and thus help doctors in the diagnosis of patients [6,7,8]. Compared to traditional endoscopes, wireless capsule endoscopes offer a non-invasive solution, has broad application prospects, and is able to avoid the occurrence of pain. However, problems still exist in that it is hard to provide the position information of the capsule corresponding to each endoscopic image and feedback which the capsule poses parameters for in further treatment operations. In recent years, scholars have done much research and proposed a variety of localization methods [9,10]. The radio frequency signal-based localization method is one of the most common methods used [11,12,13], which consists of a reader and a tag, and requires positioning targets with tags and antennae. It is not suitable for narrow environments, such as in the gastrointestinal tract. Ultrasound-based methods [14,15] have been widely used in the biomedical area, but their accuracy is limited due to speed-of-sound variations in human tissue. Medical imaging methods [16,17] have used images from the endoscope to do localizations, but they depend on the accuracy of the sample before the test, and the matching of image features is difficult to achieve. In addition, nuclear medicine imaging technology, such as CTs and MRIs, can also be used for in vivo positioning, but long-term exposure is not conducive to human health.

The magnetic localization method [18,19,20,21] was also considered as preferred technology. Here, a permanent magnet is mounted onto the capsule robot, and its position can be obtained by sensing the magnetic field of the magnet. Ref. [22] employed a novel probe to perceive the position of magnetic materials. Simple facilities and a low-cost algorithm were adapted in this approach. In addition to medical applications, magnetic positioning technology has been widely used in other fields in recent years, such as indoor mobile phone positioning [23], and automatic vehicle navigation systems [24,25].

However, the above positioning methods can only provide the absolute position information of the capsule in the tracking space. In fact, the GI tract is constantly squirming, which means a certain position in the space may correspond to different GI positions. Therefore, under the conditions of GI tract peristalsis and fluttering of the belly, the absolute tracking results may fail to provide the correct tissue position, and thus affect the correctness of the diagnosis. Some researchers proposed relative positioning methods based on the human body coordinate system [26,27]. By using additional tracking objects mounted on the human body to serve as tracking references, these methods can easily transform the capsule position results into the body coordinates. Moreover, this method can eliminate the tracking error caused by the relative movement between the human body and the tracking sensor array. However, when the GI tract is peristaltic, the proposed positioning method still cannot provide the precise position of the target in the GI tract. In addition, some medical procedures may need relative length information to determine the location of the lesion, such as endoscopy to stop bleeding, the dissection of polyps, and endoscopic biopsy. Therefore, how one can accurately reflect the matching positioning results of the capsule robot and the intestinal anatomy is an important problem for current capsule robots to be applied in clinical applications.

**Figure 1:**
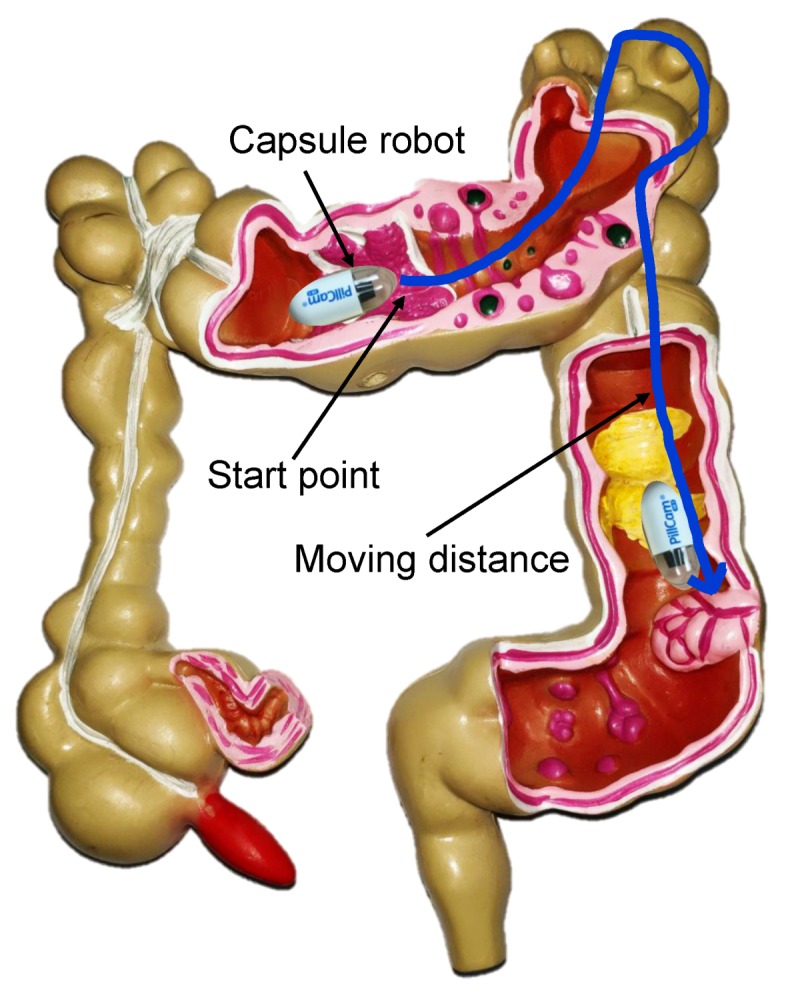
The proposed distance-based relative tracking method. The moving distance of the capsule robot relative to a marker position along the gastrointestinal (GI) tract was used to indicate the current position result.

Above all, neither the absolute localization algorithm based on the sensor coordinate system, nor the relative localization algorithm based on the human coordinate system can provide the appropriate tracking results that can be effectively used for further endoscopy operations. Some researchers have utilized the simultaneous localization and mapping (SLAM) method to obtain position information [28,29,30,31,32,33]. Usually, vision information has been used for tracking by applying the learning methods. However, the intestinal environment is full of mucus and food debris; thus, it is difficult to get the image information to match correctly. As a result, SLAM positioning may also be affected. Moreover, none of the above methods consider that the peristaltic process of the intestine causes a short retrospective process of the robot. To overcome this problem and provide a proper position description method to match the GI tract, we here propose a relative position method for tracking the movement of a capsule robot in the GI tract. As shown in Figure 1, the proposed method uses the moving distance of the robot relative to a marker position along the GI tract to indicate the current position result. First, the positioning method based on magnetic field strength was used to provide the absolute position, which could provide a high-precision, unobstructed wireless positioning result in real time [34,35,36]. Based on the absolute position, the movement path of the capsule robot was fitted with the Bézier curve. Finally, the distance information could then be estimated with the curve integration method. Compared to state-of-the-art capsule tracking methods, the proposed method can directly help guide medical instruments by providing physicians the insertion distance in patients’ bodies, which cannot be done based on position results of absolute coordinates. Moreover, as relative distance information has been used, no reference tracking objects need to be mounted onto the human body.

The structure of this paper is organized as follows: the absolute localization method based on the magnetic field is illustrated in Section 2. The algorithms for direction prediction, judgment, and moving distance calculations are developed in Section 3. Section 4 describes the experiments used to verify the accuracy of the proposed method. Finally, conclusions are drawn in Section 5.

## 2. Absolute Position Estimation Method

Magnetic tracking is the most widely studied method for wireless capsule robots. The magnetic permeability of the human body is almost the same as that of air, and the distribution of the static magnetic field is basically unaffected by human tissue. The volume of the permanent magnet can be small and easily embedded in the capsule robot, which provides conditions for miniaturization of the capsule robot. In addition, the field strength of the permanent magnet is stable, and no additional energy or peripheral circuitry is required; therefore, wireless tracking can be achieved. Due to the above advantages, the magnetic tracking method was used for the absolute positioning of wireless capsule robots in this paper.

**Figure 2:**
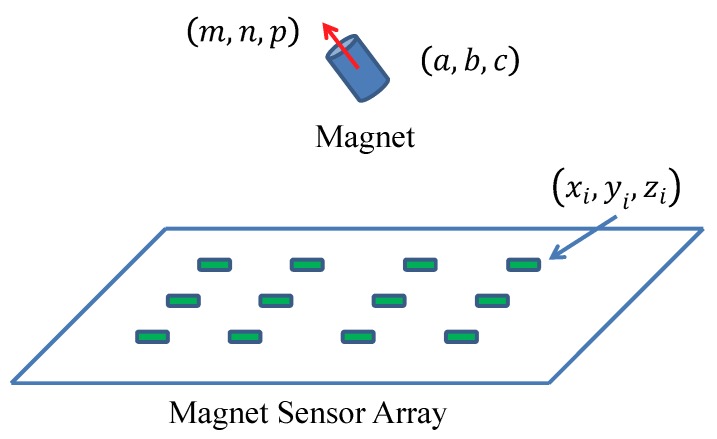
Magnetic tracking system. When a small magnet moves above a magnetic sensor array, its position ${\left(a,b,c\right)}^{T}$ and orientation ${\left(m,n,p\right)}^{T}$ can be estimated in real time.

As shown in Figure 2, the magnetic tracking system contains two main parts—a magnet, and a magnetic sensor array. The magnet can be embedded in a capsule robot to provide a magnetic field, which can be measured by the sensor array. By minimizing the difference between the sensing field from magnetic sensors and the theoretic field from the magnetic field model, the position ${\left(a,b,c\right)}^{T}$ and orientation ${\left(m,n,p\right)}^{T}$ of the capsule robot can be obtained using the optimization method.

As shown in Figure 2, the position of the *i*th sensor is ${\left(x_{i},y_{i},z_{i}\right)}^{T}$, where (i=1,2,...,N), and its theoretical magnetic field (that generated by the magnet) can be estimated based on the magnetic dipole model, as shown in the following equation:

(1) $$\begin{array}{cc} B_{i} & =B_{ix}i+B_{iy}j+B_{iz}k\\ & =\frac{\mu_{r}\mu_{0}M_{T}}{4\pi }\left(\frac{3\left(H_{0}\cdot P_{i}\right)P_{i}}{R_{i}^{5}}-\frac{H_{0}}{R_{i}^{3}}\right) \end{array}$$

where ${\left(B_{ix},B_{iy},B_{iz}\right)}^{T}$ are the three components of the magnetic field parallel to the coordinate axis, $\mu_{r}$ is the relative permeability of air, $\mu_{0}(=4\pi$ × $10^{-7}T\cdot m/A)$ is vacuum permeability, $M_{T}$ (with unit $A\cdot m^{2})$ is a constant characterizing the magnet, which relates to its size and material, and $P_{i}$ and $R_{i}$ are defined as follows:

$$P_{i}={\left(x_{i}-a,y_{i}-b,z_{i}-c\right)}^{T}$$

$$R_{i}=\sqrt{{\left(x_{i}-a\right)}^{2}+{\left(y_{i}-b\right)}^{2}+{\left(z_{i}-c\right)}^{2}},$$

$H_{0}={\left(m,n,p\right)}^{T}$ is a normalized vector characterizing the direction of the magnetic moment of the magnet and under the constrain:

(2) $$m^{2}+n^{2}+p^{2}=1.$$

The error function is defined as follows:

(3) $$\varepsilon_{i}={∥V_{i}-B_{i}∥}_{2}^{2},$$

where $V_{i}={\left(V_{xi},V_{yi},V_{zi}\right)}^{T}$ is the measured value from the *i*th magnetic sensor, and $B_{i}={\left(B_{xi},B_{yi},B_{zi}\right)}^{T}$ is the estimated value based on the magnetic field model. The magnetic localization system estimates the unknown state vector $x={\left(a,b,c,m,n,p\right)}^{T}$ of the magnet by minimizing the accumulative error function $f_{err}$,

(4) $$\begin{array}{c} \hat{x}=\underset{x}{\arg\min}\;f_{err}\;, \end{array}$$

(5) $$\begin{array}{c} f_{err}=\sum_{i}\varepsilon_{i}\;, \end{array}$$

where $\hat{x}$ is the estimated state vector of the magnet. The position and orientation ${\left(a,b,c,m,n,p\right)}^{T}$ of the magnet can then be estimated by using the optimization method.

## 3. The Relative Position Tracking Method

The movement of the capsule robot in the GI tract is a complex and irregular process. Its position and orientation information is related to the movement of the body, the vibration of the belly, and the motility of the GI tract. For the positioning problem when the human body moves, a positioning method based on the human body coordinate system was proposed in our previous work [27]. However, the problem of evaluating the distance of a capsule robot relative to a marker point based on the absolute position of the space with squirming of the GI is necessary for medial surgery, but has not been well-solved. The proposed method uses the length of the moving path between the current location and the landmark along the GI tract as the relative position of the robot. The proposed method consists of four steps: Firstly, effective sampling points will be obtained from the absolute tracking results. Secondly, the moving direction of the capsule robot will be evaluated by adjacent sampling points. Curve-fitting and integrating methods will then be performed with different direction paths to obtain the curve length. Finally, the length of the moving path can be estimated by superimposing or subtracting the lengths of the curves with different directions, respectively.

### 3.1. Moving States of the Robot

**Figure 3:**
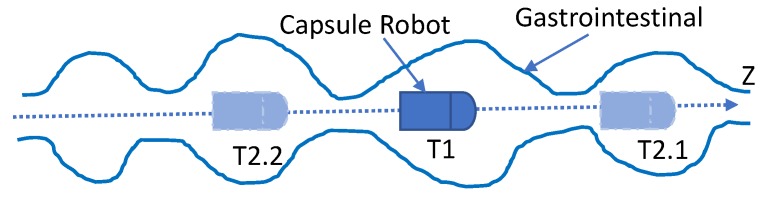
The state of the capsule robot during squirming of the GI. *Z* is the direction of the exit. $T_{1}$ means the current position of the capsule robot. $T_{2.1}$ denotes the next possible position when the robot is moving forward, and $T_{2.2}$ denotes the next possible position when the robot is moving backward.

The movement of the capsule robot in the human body is mainly driven by peristalsis of the gastrointestinal tract. The basic mechanical movement of the intestine consisting of a contracted wavy ring was recognized by most people, which can be seen in Figure 3. The motility of the gastrointestinal tract is mainly to push the object that is moving forward (toward the direction of the exit). However, due to the contraction of the contracted wavy, the object behind the contracted wave would have a small direction of reversal. As shown in Figure 3, the rectangular blocks represent the capsule robot. The wavy curves indicate the intestine and its wave ring. *Z* is the direction of the exit, $T_{1}$ means the position of the capsule robot at present, and $T_{2.1}$ and $T_{2.2}$ denote the next possible position due to peristalsis of the GI tract. According to this situation, the moving direction of the capsule robot needs to be determined, and then the moving distance of the capsule robot relative to a certain feature point can be accumulated by increasing or decreasing the distance length.

**Figure 4:**
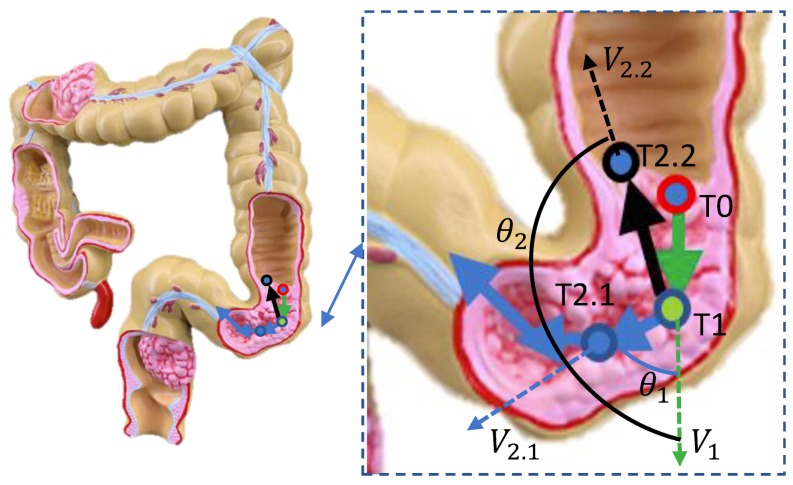
The state of the capsule robot moving in the corner. *P* shows the current position of the capsule robot, and $V_{1}$ reveals the robot’s movement at the last moment. The robot may move in the direction of $V_{2.1}$ or $V_{2.2}$ at the next moment, and the angle was formed by the adjacent moments $\theta_{1}$ and $\theta_{2}$, respectively.

Although the GI twines around the human body, it also has some corners. Therefore, the angle between two adjacent moving direction vectors may vary within a certain range. Figure 4 describes the moment of capsule robots moving in the corner of the GI. As shown in Figure 4, at the next moment of point *P*, the capsule robot may move in direction $V_{2.1}$ or $V_{2.2}$. It is assumed that moving in the direction of $V_{2.2}$ is normally to go forward. However, when moving in the direction of $V_{2.1}$, the capsule robot goes backward. The angle between two adjacent moving direction vectors can be estimated as follows:

(6) $$\theta =arccos\frac{V_{1}\cdot V_{2}}{∥V_{1}-V_{2}∥},$$

where $V_{1}$ is the vector from the position of the capsule robot at point $T_{0}$ to the position of $T_{1}$. $V_{2}$ is the vector from the position of $T_{1}$ to $T_{2}$. Therefore, we can have:

(7) $$V_{1}={\left(T_{1x}-T_{0x},T_{1y}-T_{0y},T_{1z}-T_{0z}\right)}^{T}$$

(8) $$V_{2}={\left(T_{2x}-T_{1x},T_{2y}-T_{1y},T_{2z}-T_{1z}\right)}^{T}$$

In addition, the positioning error of the magnetic sensor array we used was around 1.2 mm, which is shown in the experimental section. Therefore, to reduce the interference from the tracking result and obtain a smoother path, when the distance *l* from the last point to the current point was smaller than 4 mm, the position of the capsule robot was assumed to be unchanged, where

$$l=\sqrt{{\left(T_{1(x)}-T_{0(x)}\right)}^{2}+{\left(T_{1(y)}-T_{0(y)}\right)}^{2}+{\left(T_{1(z)}-T_{0(z)}\right)}^{2}}.$$

In this paper, the reverse of the capsule robot is considered when θ ∈ $\left[150^{\circ },210^{\circ }\right]$. The particular angle in our consideration was only used as a proof of concept, and with more advanced technology in the gastrointestinal kinematics model, we would have been able to define θ in more detail, considering that the distance between adjacent sampling points is only slightly larger than 4 mm. When the robot moves forward along the corner, the sampling points will not be reversed instantaneously, but slowly distributed along the curve. On the contrary, the reversal caused by the contraction of the gastrointestinal tract will lead to the instantaneous reversal of sampling points. According to a large number of gastrointestinal model tests, we believe that θ ∈ $\left[150^{\circ },210^{\circ }\right]$ can distinguish whether the capsule robot is turning forward or moving backwards.

### 3.2. Segmentation Processing

As mentioned in the section above, the capsule robot generally moves towards the exit, but may sometimes reverse due to the squirming of the GI tract. Therefore, for calculating the moving distance of the capsule robot, different moving directions require a different approach.

**Figure 5:**
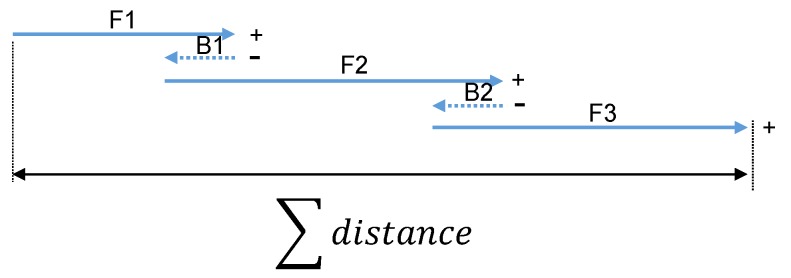
An example of a moving sequence of the capsule robot. $F_{1}$, $F_{2}$, and $F_{3}$ represent the capsule robot’s movements forward, and $B_{1}$ and $B_{2}$ represent the process of the robot’s movements backward.

Figure 5 shows a moving sequence of the capsule robot. $F_{1}$, $F_{2}$ and $F_{3}$ represent how the capsule robot was moving forward, and $B_{1}$ and $B_{2}$ represent the process of a short reverse of the capsule robot.

A moving directional flag (D.Flag) was set in the experimental program in order to provide the direction information of the micro-robot in real time. The D.Flag was initialized to 1, and transferred to −1 when the direction changed, which repeated during the whole examination progress. $D.Flag={\left(-1\right)}^{n}$, where *n* represents the changing times of the moving direction.

In this part, $F_{1}$, $F_{2}$, and $F_{3}$ increase the moving length, but $B_{1}$ and $B_{2}$ decrease the whole distance. Therefore, the moving distance of the capsule robot under this sequence is calculated as:

(9) $$d=F_{1}+F_{2}+F_{3}-B_{1}-B_{2}.$$

Then, the moving length of the capsule robot in the GI can be estimated as follows:

(10) $$L=\sum_{i=1}^{m}F_{m}-\sum_{i=1}^{n}B_{n},$$

where $F_{m}$ is *m*-th distance of the capsule robot moving forward, and $B_{n}$ is the *n*-th distance of the capsule robot moving backward.

The swimming speed of micro-robot can also be obtained during the examination. In each period, the average swimming speed can be calculated as $\bar{V}=S/T$, where *S* means the moving length, and *T* indicates the swimming time. In order to obtain instantaneous speed, the period should be divided into a smaller part.

### 3.3. Moving Distance

**Figure 6:**
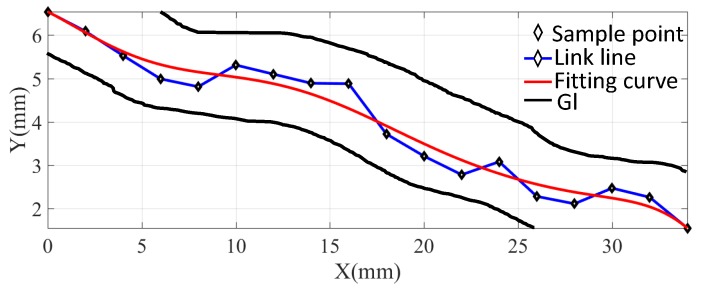
The state of the capsule robot during squirming of the GI. The blue stars represent possible sampled position results. The red line is the curve-fitting result. The black lines represent the GI tract. It can be seen that the red line matches the length of the GI better than the blue line.

The above distance estimation method can have a distance estimation, but it still has drawbacks. The intestinal diameter of an adult is about 20–40 mm, while the diameter of a capsule is about 12 mm. This means that the capsule robot may shake backwards and forwards in one place, which can be seen in Figure 6.

As shown in Figure 6, blue stars are the possible sampled position results. The robot’s shaking may cause the length of the blue line to be longer than the actual length of the GI tract. Therefore, to minimize the error of distance accumulation and better match the moving length of the capsule robot, the Bézier curve was used to fit the sampling points to make the path smoother. As shown in Figure 6, the red line is the curve-fitting result. It can be seen that the red line matches the length of the GI better than the blue line.

The Bézier curve can be seen to produce a smooth curve that reduces distance error due to oscillation. In this paper, every section of $F_{m}$ and $B_{n}$ was fitted by a piece of a quadratic Bézier curve. The length of each curve was then calculated by integrating each fitting curve. Finally, the moving length of the capsule robot was gained by adding the distance of moving toward the exit and subtracting the distance moved backward.

The general form of the quadratic Bézier curve is presented as follows:

(11) $$B_{t}={\left(1-t\right)}^{2}P_{0}+2t\left(1-t\right)P_{1}+t^{2}P_{2},$$

where t∈ $\left[0,1\right]$. For each Bézier curve, positions of each point $P_{i}$, (i=0,1,2) can be obtained from the absolute tracking results.

Therefore, each segment length can be obtained by integrating $B_{t}$, which is:

(12) $$distance=\int_{P_{0}}^{P_{2}}B_{t}dt.$$

The final moving length of the capsule robot can be gained by adding each length of the Bézier curve, which is:

(13) $$L=\sum \left(distance_{+}\right)-\sum \left(distance_{-}\right)$$

where $distance_{+}$ is the length of the capsule robot moving forward, while $distance_{-}$ is the length of the robot moving backward.

According to the above calculation process, the algorithm flow diagram is shown in Algorithm 1.

**Algorithm 1** Distance calculation for the moving capsule.**Input:** Absolute position $P_{i}=\left(x_{i},y_{i},z_{i}\right)$ **Output:** Moving distance *L*1:set flag=02:set L=03:**repeat**4: Get absolute position $P_{i}$5: **if** $\left|P_{i}-P_{i-1}\right|\geq 4$ mm **then**6:  Estimate adjacent moving angle θ7:  **if** $\theta \in \left[150^{\circ },210^{\circ }\right]$ **then**8:   flag=flag⊕19:  **end if**10:  Quadratic Bézier curve-fitting11:  $distance=\int_{P_{0}}^{P_{2}}B_{t}dt$12:  $L=L+distance\times {\left(-1\right)}^{flag}$13: **else**14:  $P_{i}$ = $P_{i-1}$15: **end if**16:**until** Tracking End17:**return***L*

## 4. Experiments and Results

### 4.1. Static Model Experiments

**Figure 7:**
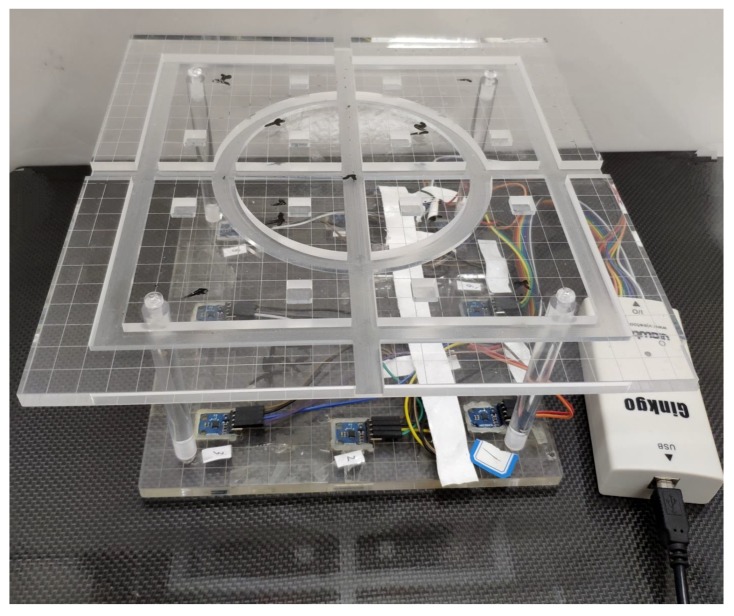
Experimental platform. The magnetic sensors array contained 8 HMC5883L sensors. Signals from the sensors were transferred to a PC via an IIC to a USD adapter. A plane with a circle path and rectangular path was placed above the sensor array to carry out the test.

To verify the proposed method, static environmental experiments were carried out to test the measurement accuracy under different environments and conditions. Figure 7 shows the magnetic tracking system. A plane with a circle path and rectangular path was placed above the sensor array to carry out the test. The magnetic tracking system had a position error of 1.2 mm and orientation error of $3.2^{\circ }$ according to our test before the experiments.

**Figure 8:**
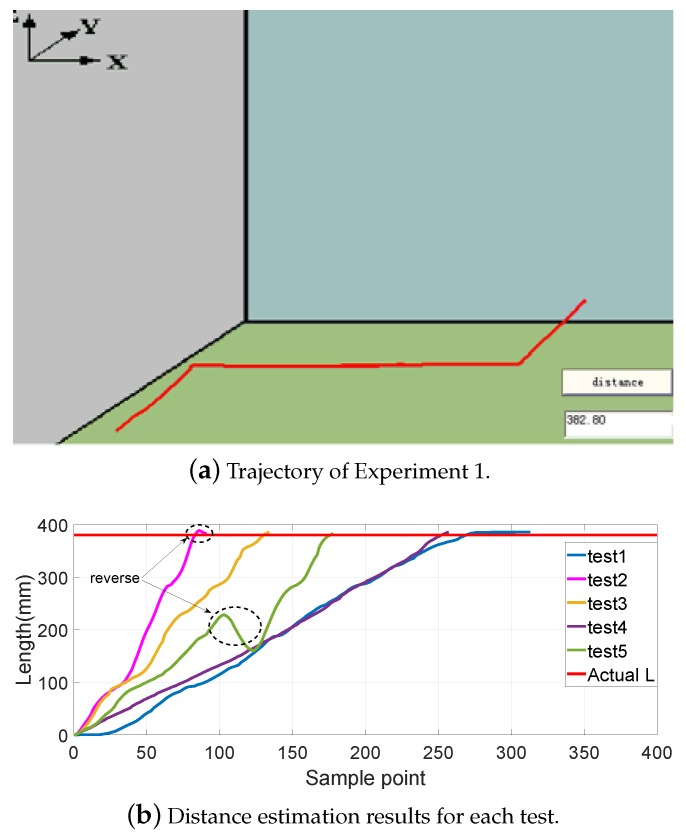
Experiment 1: a Z-shape track with a length of 380 mm.

**Figure 9:**
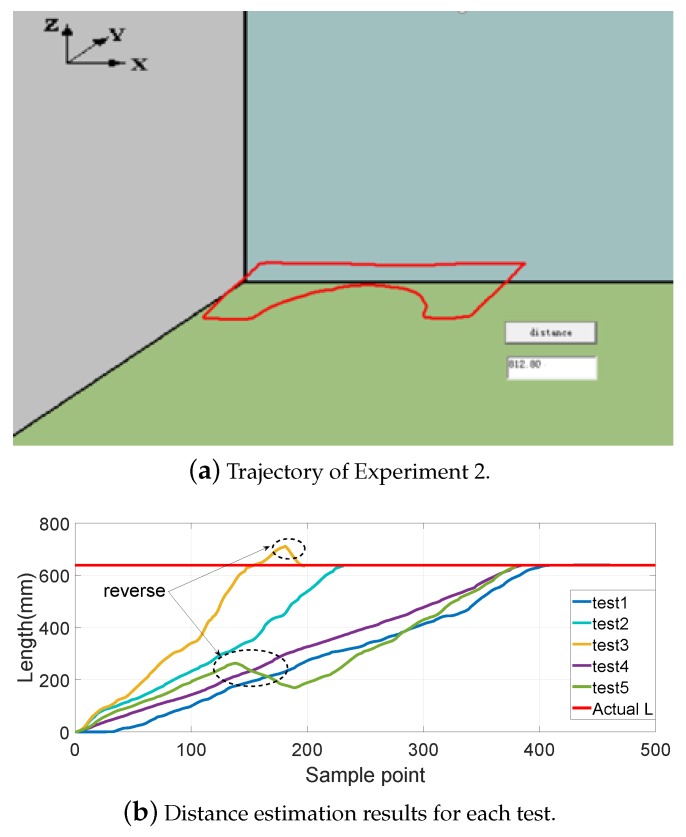
Experiment 2: consisting of straight lines and a semicircular arc; path length is 638 mm.

**Figure 10:**
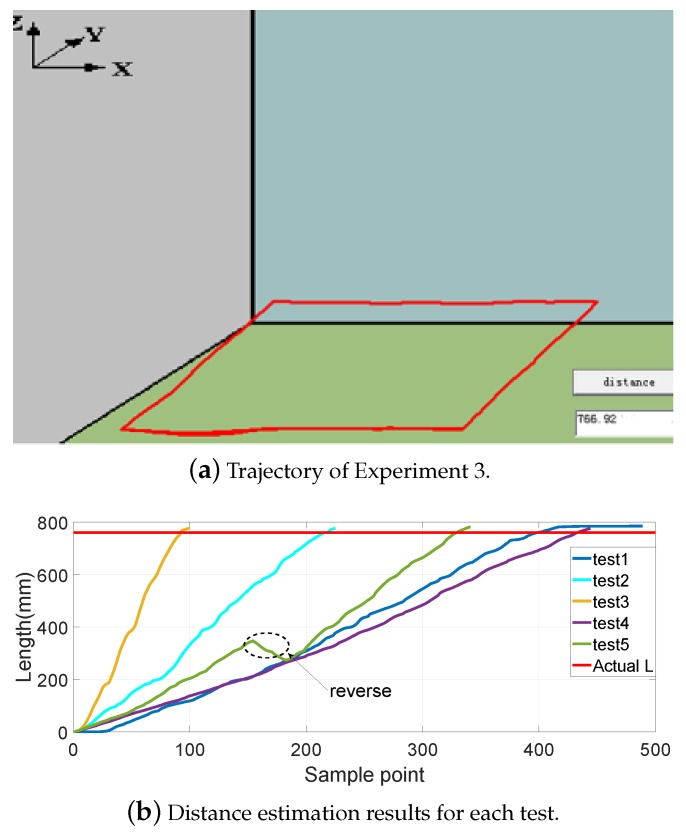
Experiment 3: a square path consisting of four straight lines with a length of 190 mm; total path length 760 mm.

**Figure 11:**
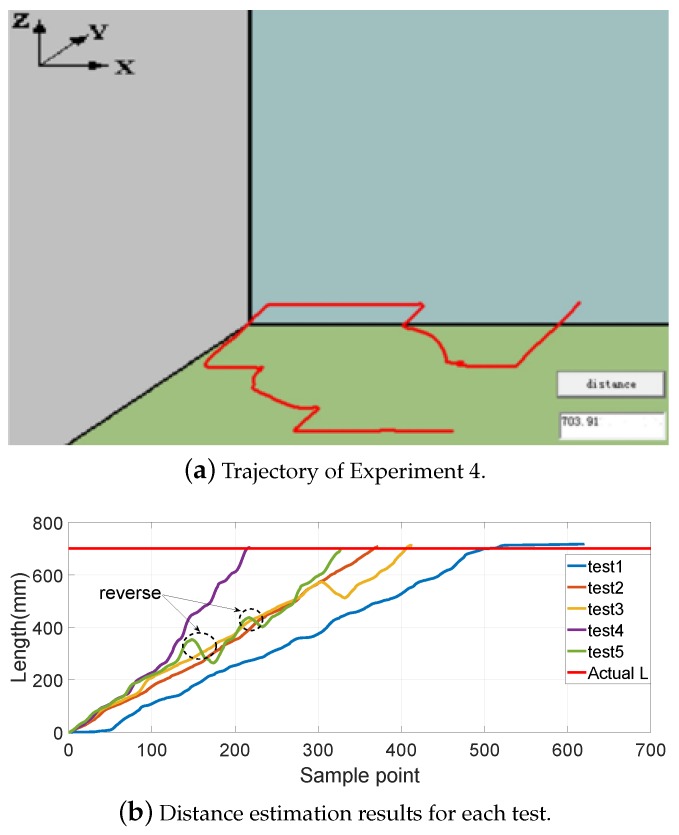
Experiment 4: includes two arcs and several straight lines, total length being 700 mm.

Figure 8, Figure 9, Figure 10 and Figure 11 show different distance estimation results in the regular grooves model under different paths. Figure 8 is a Z-shape track, whose length is 380 mm. Figure 9 consists of straight lines and a semicircular arc, and its path length is 638 mm. Figure 10 shows a square path that consists of four straight lines with a length of 190 mm, and the total path length is 760 mm. Figure 11 includes two arcs and several straight lines, the total length being 700 mm. For each experiment, five tests with different speeds and disturbances were performed. The red lines in each sub-figure (b) represent the real-time trajectory tracking results of the capsule robot in 3D space. Real-time distance estimation straight results are shown in the bottom-right of the interface. For each of the sub-figures (a), lines with different colors represent the different moving speeds of the magnet. Some forward- and backward-moving disturbances were also added to some trails, which can be seen in figures with circle markers. A horizontal straight line represents the ground-truth length of the track. As shown in the figures, the distance values are all larger than the real value at the end of each curve. This was caused by the shaking of the magnet while moving during the experiments.

**Figure 12:**
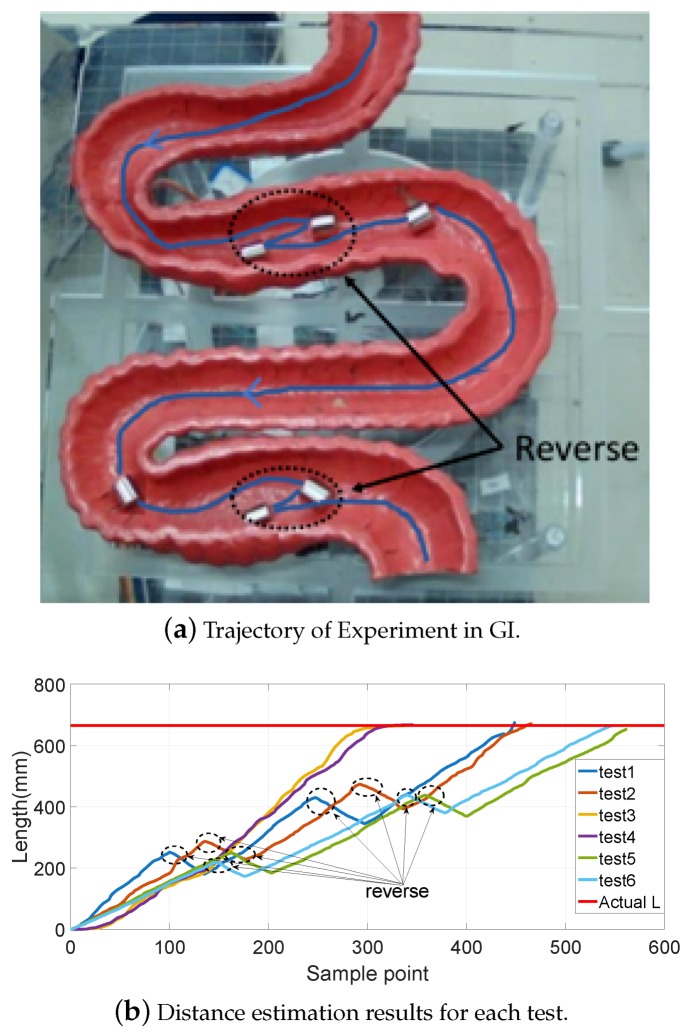
Experiment of the GI tract.

Moreover, the static GI tract model experiments were also carried out, as shown in Figure 12a. The length of the model along the central line was approximately 665 mm. The movement passing through the model was performed six times with different speeds and interferences, and the results are shown in Figure 12b. To better simulate the process of the capsule robot moving in the GI, shakings left and right and forward and backward movements were also added during the experiments.

**Figure 13:**
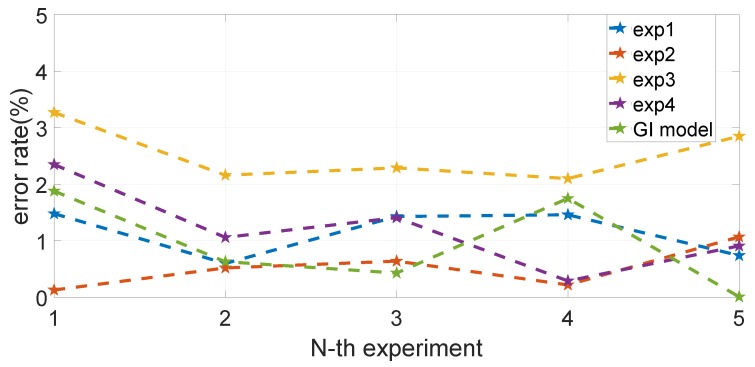
Experimental error results. The maximum distance error is 3.3%, and the final average is 1.27%, which is acceptable for further endoscopy insertion operations.

Table 1 and Table 2, as well as Figure 13 show the distance measurement errors of the capsule robot in different trajectories. The error is defined as follows:

(14) $$Err=\frac{|D_{est}-D_{gt}|}{D_{gt}}\times 100\%,$$

where $D_{est}$ is the estimated distance, and $D_{gt}$ is the ground truth. It can be seen that there is no accumulated error with increasing measuring distance. The maximum distance error is 3.3%, and the final average is 1.27%, which is acceptable for further endoscopy insertion operations.

### 4.2. Dynamic Phantom Experiments

**Figure 14:**
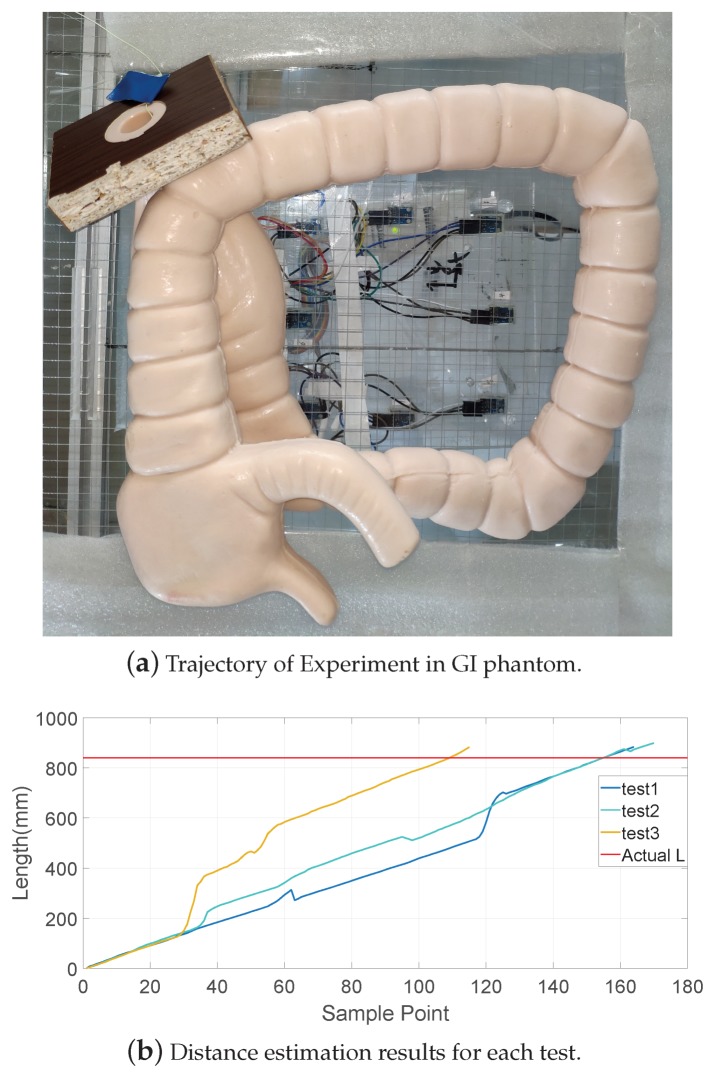
Experiment in a GI phantom. In each test, the robot was placed at a depth of 840 mm from the exit, and then carried out by dragging with a rope. In this experiment, the length of the rope acted as ground truth, which was then compared with the accuracy of the magnetic positioning system to judge the reliability of our system.

In order to simulate a more realistic dynamic environment of the capsule robot’s moving state in the GI tract, an artificial intestinal phantom with a layer of diseased tissue, such as polyps and tumors made of silicone, was tested in the experiments. As shown in Figure 14a, at the exit of the GI tract, a rope was used to drag the robot for the purpose of simulating the robot’s moving state caused by peristalsis of the human intestine, and all led to the robot’s overall trend of moving forward. At the beginning of the movement, a blue marker was tied onto the rope, and the experiment stopped when the robot came out of the intestine. The length from the marker of the rope to the end when the robot came out acted as ground truth. Figure 14b shows the experimental results in the GI. The red horizontal line is the depth at where the robot was placed acted as ground truth, which was 840 mm. The distance estimation experiments were carried out three times, and the tracking results were 883.6 mm, 898.8 mm, and 882.6 mm, respectively. The average error was 5.7%. The results are highly close to the ground truth, but there was still some fluctuation. This was caused by the existence of certain errors in the positioning system itself, since the moving positions were mainly on the border of the tracking system, where the positioning error was slightly larger.

### 4.3. Comparison

Some articles have also researched the movement path of capsule robots [28,29,30,31,32,33]. The visual odometer, SLAM, and machine learning methods were adopted in the above article. A comparison between the experimental results of this paper and these methods is shown in Table 3. It can be seen that the proposed method achieved good performance.

Compared to the above methods, the proposed method was subjected to less interference. Since the intestinal environment was filled with mucus and physical debris and the intestines were creeping about in real time, methods using image information based on a static intestinal environment may fail to provide available results in clinical applications. However, the proposed method still has limitations, in that the magnetic tracking system has a small sensing distance. For real clinical applications, a larger sensor array with more sensors would be needed.

## 5. Conclusions

Although positioning and tracking algorithms and techniques of capsule robots have been extensively studied, but there is still a lack of methods that can provide position results that match with the anatomy of the GI. In this paper, a method for measuring the movement length of a capsule robot in the human body has been proposed. The magnetic tracking system and curve-fitting method were used to obtain the moving distance result. Experimental results verified the proposed method. A maximum error of 3.3% was obtained. In the future, we will continue to improve the proposed method and further carry out animal experiments.

## Figures and Tables

**Table 1:** Distance estimation results with different experiments.

Item	$T_{1}$ (mm)	$T_{2}$ (mm)	$T_{3}$ (mm)	$T_{4}$ (mm)	$T_{5}$ (mm)	Ground Truth (mm)
Experiment 1	386	382	385	386	383	380
Experiment 2	639	635	634	637	632	639
Experiment 3	785	776	777	776	782	760
Experiment 4	717	708	711	703	694	701
GI model	677	669	668	653	665	665

**Table 2:** Experimental error results.

Item	Average Error	Max Error
Experiment 1	1.16%	1.58%
Experiment 2	0.56%	1.1%
Experiment 3	2.53%	3.3%
Experiment 4	1.20%	2.28%
GI model	0.93%	1.8%
Final	1.27%	3.3%

**Table 3:** Comparisons between the proposed method and state-of-the-art methods.

Methods	Error Rate
Proposed method in static environment	1.27%
Proposed method in dynamic phantom	5.7%
Large-scale direct monocular (LSD) SLAM [31]	around 14.0%
Oriented fast and rotated brief (ORB) SLAM [32]	around 13.2%
Magnetic localization [29]	around 6.8%
Visual localization [33]	around 6.5%
Fusion by deep visual and magnetic [37]	around 4.3%
Unsupervised visual Odometry and Depth Learning [30]	around 6.2%

## References

[1] World Health Organization. http://www.who.int/zh/news-room/fact-sheets/detail/cancer/.

[2] Levin B., Lieberman D.A., McFarland B., Smith R.A., Brooks D., Andrews K.S., Dash C., Giardiello F.M., Glick S., Levin T.R. (2008). Screening and surveillance for the early detection of colorectal cancer and adenomatous polyps, 2008: A joint guideline from the American Cancer Society, the US Multi-Society Task Force on Colorectal Cancer, and the American College of Radiology. CA Cancer J. Clin..

[3] Valdastri P., Simi M., Webster R.J. (2012). Advanced technologies for gastrointestinal endoscopy. Ann. Rev. Biomed. Eng..

[4] Leung B.H., Poon C.C., Zhang R., Zheng Y.L., Chan C.K., Chiu P.W., Lau J.Y., Sung J.J. (2016). A Therapeutic Wireless Capsule for Treatment of Gastrointestinal Haemorrhage by Balloon Tamponade Effect. IEEE Trans. Biomed. Eng..

[5] Yu W., Rahimi R., Ochoa M., Pinal R., Ziaie B. (2015). A smart capsule with GI-tract-location-specific payload release. IEEE Trans. Biomed. Eng..

[6] Song S., Qiu X., Wang J., Meng M.Q.H. (2016). Real-time tracking and navigation for magnetically manipulated untethered robot. IEEE Access.

[7] Keller H., Juloski A., Kawano H., Bechtold M., Kimura A., Takizawa H., Kuth R. Method for navigation and control of a magnetically guided capsule endoscope in the human stomach. Proceedings of the 2012 4th IEEE RAS & EMBS International Conference on Biomedical Robotics and Biomechatronics (BioRob).

[8] Winstone B., Melhuish C., Pipe T., Callaway M., Dogramadzi S. (2017). Toward Bio-Inspired Tactile Sensing Capsule Endoscopy for Detection of Submucosal Tumors. IEEE Sens. J..

[9] Mateen H., Basar M.R., Ahmed A.U., Ahmad M.Y. (2017). Localization of Wireless Capsule Endoscope: A Systematic Review. IEEE Sens. J..

[10] Nowicki M., Szewczyk R. (2019). Determination of the Location and Magnetic Moment of Ferromagnetic Objects Based on the Analysis of Magnetovision Measurements. Sensors.

[11] Reza A.W., Yun T.W., Dimyati K., Tan K.G., Noordin K.A. (2012). Deployment of a 3D tag tracking method utilising RFID. Int. J. Electron..

[12] Tesoriero R., Gallud J.A., Lozano M.D., Penichet V.M. (2009). Tracking autonomous entities using RFID technology. IEEE Trans. Consum. Electron..

[13] Vitas I., Zrno D., Simunic D., Prasad R. Innovative RF localization for wireless video capsule endoscopy. Proceedings of the 2014 ITU Kaleidoscope Academic Conference: Living in A Converged World-impossible Without Standards.

[14] Diamantis K., Dermitzakis A., Hopgood J.R., Sboros V. (2018). Super-resolved ultrasound echo spectra with simultaneous localization using parametric statistical estimation. IEEE Access.

[15] Diamantis K., Anderson T., Jensen J.A., Dalgarno P.A., Sboros V. (2019). Development of Super-resolution Sharpness-based Axial Localization for Ultrasound Imaging. IEEE Access.

[16] Costamagna G., Shah S.K., Riccioni M.E., Foschia F., Mutignani M., Perri V., Vecchioli A., Brizi M.G., Picciocchi A., Marano P. (2002). A prospective trial comparing small bowel radiographs and video capsule endoscopy for suspected small bowel disease. Gastroenterology.

[17] Yu F., Song E., Liu H., Zhu J., Hung C.C. (2019). Laparoscopic Image-Guided System Based on Multispectral Imaging for the Ureter Detection. IEEE Access.

[18] Song S., Qiu X., Liu W., Meng M.Q.H. (2017). An Improved 6-D Pose Detection Method Based on Opposing-Magnet Pair System and Constraint Multiple Magnets Tracking Algorithm. IEEE Sens. J..

[19] Qiu X., Song S., Meng M.Q.H. (2017). A novel 6-D pose detection method using opposing-magnet pair system. IEEE Sens. J..

[20] Dai H., Song S., Zeng X., Su S., Lin M., Meng M.Q.H. (2018). 6-D electromagnetic tracking approach using uniaxial transmitting coil and tri-axial magneto-resistive sensor. IEEE Sens. J..

[21] Song S., Li B., Qiao W., Hu C., Ren H., Yu H., Zhang Q., Meng M.Q.H., Xu G. (2014). 6-D magnetic localization and orientation method for an annular magnet based on a closed-form analytical model. IEEE Trans. Magn..

[22] Yanus R., Vedenev M., Drozhzhina V., Reutov Y.Y. (1967). Use of magnetic pole-finding probe in surgical extraction of magnetic foreign bodies. Biomed. Eng..

[23] Kasmi Z., Norrdine A., Blankenbach J. (2015). Towards a decentralized magnetic indoor positioning system. Sensors.

[24] Su S., Zeng X., Song S., Lin M., Dai H., Yang W., Hu C. (2018). Positioning Accuracy Improvement of Automated Guided Vehicles Based on a Novel Magnetic Tracking Approach. IEEE Intell. Transp. Syst. Mag..

[25] Mitterer T., Gietler H., Faller L.M., Zangl H. (2019). Artificial Landmarks for Trusted Localization of Autonomous Vehicles Based on Magnetic Sensors. Sensors.

[26] Song S., Hu C., Meng M.Q.H. (2016). Multiple Objects Positioning and Identification Method Based on Magnetic Localization System. IEEE Trans. Magn..

[27] Hu C., Ren Y., You X., Yang W., Song S., Xiang S., He X., Zhang Z., Meng M. (2016). Locating Intra-Body Capsule Object by Three-Magnet Sensing System. IEEE Sens. J..

[28] Turan M., Almalioglu Y., Araujo H., Konukoglu E., Sitti M. (2018). Deep endovo: A recurrent convolutional neural network (rcnn) based visual odometry approach for endoscopic capsule robots. Neurocomputing.

[29] Turan M., Almalioglu Y., Gilbert H., Sari A.E., Soylu U., Sitti M. (2017). Endo-VMFuseNet: deep visual-magnetic sensor fusion approach for uncalibrated, unsynchronized and asymmetric endoscopic capsule robot localization data. arXiv.

[30] Turan M., Ornek E.P., Ibrahimli N., Giracoglu C., Almalioglu Y., Yanik M.F., Sitti M. Unsupervised odometry and depth learning for endoscopic capsule robots. Proceedings of the 2018 IEEE/RSJ International Conference on Intelligent Robots and Systems (IROS).

[31] Engel J., Schöps T., Cremers D. (2014). LSD-SLAM: Large-scale direct monocular SLAM. Proceedings of the European Conference on Computer Vision.

[32] Mur-Artal R., Montiel J.M.M., Tardos J.D. (2015). ORB-SLAM: A versatile and accurate monocular SLAM system. IEEE Trans. Robot..

[33] Turan M., Almalioglu Y., Araujo H., Konukoglu E., Sitti M. (2017). A non-rigid map fusion-based rgb-depth slam method for endoscopic capsule robots. arXiv.

[34] Song S., Hu C., Li B., Li X., Meng M.Q.H. (2013). An electromagnetic localization and orientation method based on rotating magnetic dipole. IEEE Trans. Magn..

[35] Gao M., Hu C., Chen Z., Zhang H., Liu S. (2010). Design and fabrication of a magnetic propulsion system for self-propelled capsule endoscope. IEEE Trans. Biomed. Eng..

[36] Natali C.D., Beccani M., Valdastri P. (2013). Real-Time Pose Detection for Magnetic Medical Devices. IEEE Trans. Magn..

[37] Turan M., Almalioglu Y., Ornek E.P., Araujo H., Yanik M.F., Sitti M. Magnetic-visual sensor fusion-based dense 3d reconstruction and localization for endoscopic capsule robots. Proceedings of the 2018 IEEE/RSJ International Conference on Intelligent Robots and Systems (IROS).

